# Sheep Movement Networks and the Transmission of Infectious Diseases

**DOI:** 10.1371/journal.pone.0011185

**Published:** 2010-06-17

**Authors:** Victoriya V. Volkova, Richard Howey, Nicholas J. Savill, Mark E. J. Woolhouse

**Affiliations:** Centre for Infectious Diseases, School of Biological Sciences, University of Edinburgh, Edinburgh, United Kingdom; Yale University, United States of America

## Abstract

**Background and Methodology:**

Various approaches have been used to investigate how properties of farm contact networks impact on the transmission of infectious diseases. The potential for transmission of an infection through a contact network can be evaluated in terms of the basic reproduction number, *R*_0_. The magnitude of *R*_0_ is related to the mean contact rate of a host, in this case a farm, and is further influenced by heterogeneities in contact rates of individual hosts. The latter can be evaluated as the second order moments of the contact matrix (variances in contact rates, and co-variance between contacts to and from individual hosts). Here we calculate these quantities for the farms in a country-wide livestock network: >15,000 Scottish sheep farms in each of 4 years from July 2003 to June 2007. The analysis is relevant to endemic and chronic infections with prolonged periods of infectivity of affected animals, and uses different weightings of contacts to address disease scenarios of low, intermediate and high animal-level prevalence.

**Principal Findings and Conclusions:**

Analysis of networks of Scottish farms via sheep movements from July 2003 to June 2007 suggests that heterogeneities in movement patterns (variances and covariances of rates of movement on and off the farms) make a substantial contribution to the potential for the transmission of infectious diseases, quantified as *R*_0_, within the farm population. A small percentage of farms (<20%) contribute the bulk of the transmission potential (>80%) and these farms could be efficiently targeted by interventions aimed at reducing spread of diseases via animal movement.

## Introduction

Understanding the structure of contact networks is important for predicting and controlling the spread of infectious diseases [1]–[4]. One important route of transmission of infectious diseases of farm animals is the movement of livestock between farms [5]. In Britain, comprehensive, computerized movement-record keeping systems have been in place for cattle since 1998 and for sheep since 2002. The movement-record data have been used in studies of the epidemiology of a variety of diseases, for example, foot-and-mouth disease in cattle and sheep [6], bovine tuberculosis in cattle [7], [8], and scrapie in sheep [9], [10]. But the records of movements of British livestock between farms also provide a rare example of large and fully documented contact networks. In parallel with the disease-specific studies there have been a number of studies of the generic properties of livestock movement networks relating to the spread of infectious disease. These have taken two approaches to characterising the movement networks.

The first approach, adopted from generic network methodologies, is based on assessing the connectedness of a farm network through calculating the size of its giant connected component [11]–[14]. For a directed network (where the link between two nodes may be in one direction or the other or both) the giant strongly connected component (GSCC) is the largest subset of members of the network mutually reachable through a direct path; each pair of members in the GSCC is connected in both directions [11]. The giant weakly connected component (GWCC) is the largest subset of the network linked by any contact [11]. Therefore the GSCC and GWCC provide lower and upper bounds, respectively, to maximum epidemic size. The giant out-component (GOC) is the subset of the network approachable from the GSCC by a direct path [11]; therefore the GOC includes the GSCC itself and all the farms which can be reached directly from the GSCC. An increase in the size of the GSCC of British cattle farm network was reported after new regulations governing the movement of cattle in the UK were introduced between 2001 and 2003 [15]. This result implies that the potential scale of infectious disease epidemics in British cattle may have subsequently increased rather than decreased.

A second approach is based on evaluating the potential for transmission for an infection that may spread through the contact network in terms of the basic reproduction number, *R*_0_. In this context *R*_0_ is a measure of the expected average number of secondary cases generated from a single primary case introduced into a naïve population [16]. The relationship between *R*_0_ and the giant connected components of the network were discussed by Kao et al. [17]. An important distinction is that *R*_0_ is a function of the *rates* of contact of members of the network whereas the giant components are static measures of the network's connectedness [18].

For any contact network, contributions to *R*_0_ can be partitioned into a first order moment (relating to the mean contact rate of a member) and second order moments (relating to the variances and co-variances in contact rates of individual members) [19]. Earlier work on livestock movements and other networks (e.g. human sexual contacts) has focused on the contribution of the variance in contact rates and, for networks with bi-directional links, the covariance between contact rates in either direction [2], [14], [17], [20]. Using these measures and a sample of the cattle movement network in Scotland, Woolhouse et al. [20] concluded that the cattle network was consistent with the ‘20–80’ rule, which states that 20% of the population contribute at least 80% of the magnitude of *R*_0_ [2]. Interventions targeted at these farms could therefore be particularly effective in reducing the size of epidemics or the level of endemic infection.

Here, we analyse the entire contact network of Scottish farms via movements of sheep during 4 years from July 2003 to June 2007. Given knowledge of the complete network for each year, we calculate the sizes of GWCC, GSCC and the giant out-component, and the relative magnitude of the basic reproduction number. We partition the latter in terms of the contributions of the first and second order moments of the network. These calculations allow us to identify which features of the farm network structure, and which individual farms contribute the most to the potential for spread of infections through the network, and how these have changed from 2003 to 2007. We do not focus here on specific infections. However, because we consider a one-year time span and do not attempt to capture the early dynamics of disease outbreaks, our results are most directly relevant to endemic and chronic infections, with prolonged periods of infectivity of affected animals. By weighting differently the contacts between farms, we address diseases with three distinct scenarios of animal-level prevalence (high prevalence, low prevalence, and intermediate prevalence).

## Results

### Descriptive network statistics and network's connectedness

Descriptive statistics for the Scottish sheep farm network for the 4 years from July 2003 to June 2007 are given in Table 1. In summary, each year the number of farms in the network, *N*, was greater than 15,000, with approximately 70,000 uni-directional connections between the farms. Over 100,000 sheep batches were moved within the network per year, totalling more than 2,000,000 sheep. Approximately half of the farms that recorded moving sheep within Scotland each year were part of the GSCC, two-thirds were part of the giant-out component, and over 98% were part of the GWCC of the year's network (Table 1). The size of the GWCC confirmed that the farm network was highly inter-connected; the size of the giant out-component showed that a long-lasting infection introduced into this farm population within a year could directly reach nearly 70% of the farms via the movements of sheep.

**Table 1:** Summary statistics for the network of Scottish farms via sheep movements.

Year	Number of farms	Number of uni-directional contacts between farms	Number of sheep batches moved between farms	Number of sheep moved between farms	Size of giant strongly connected component (fraction of farms)	Size of giant out-component (fraction of farms)	Size of giant weakly connected component (fraction of farms)
Year 1	15,788	72,067	116,973	2,217,940	0.516	0.670	0.989
Year 2	15,314	71,999	118,957	2,118,099	0.505	0.666	0.989
Year 3	15,762	68,952	108,978	2,162,764	0.486	0.651	0.986
Year 4	15,750	68,347	105,500	2,266,971	0.491	0.669	0.986

**Figure 1:**
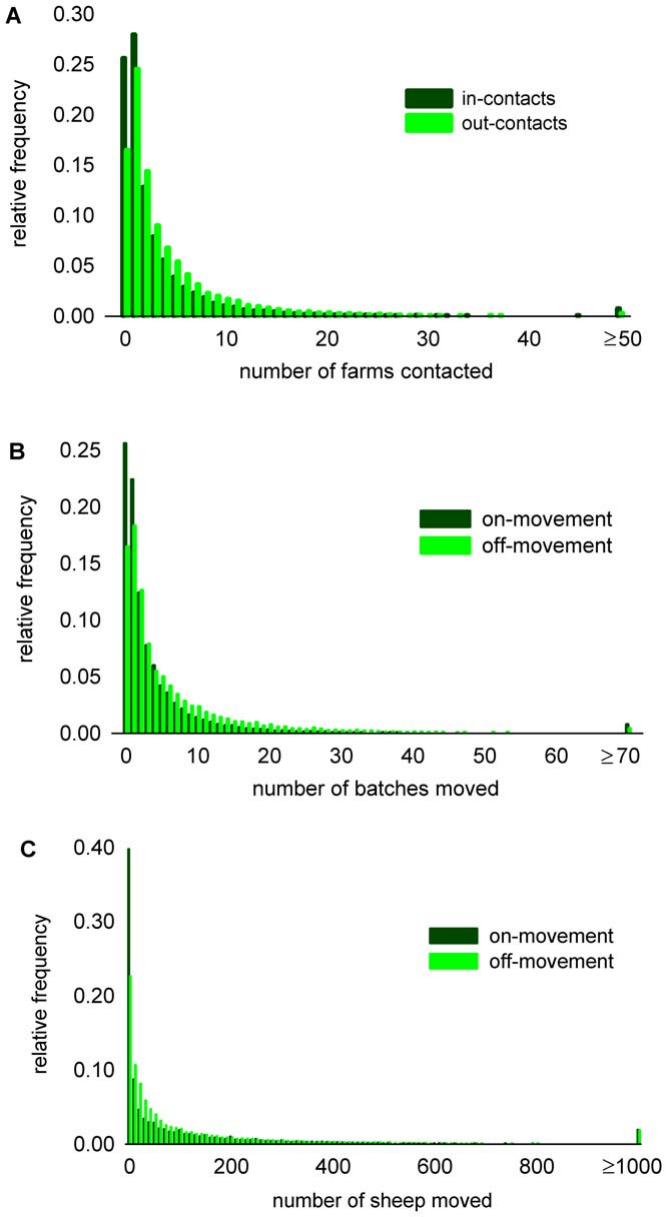
Distribution of contacts of Scottish farms via sheep movements 1 July 2006 to 30 June 2007.

**Figure 2:**
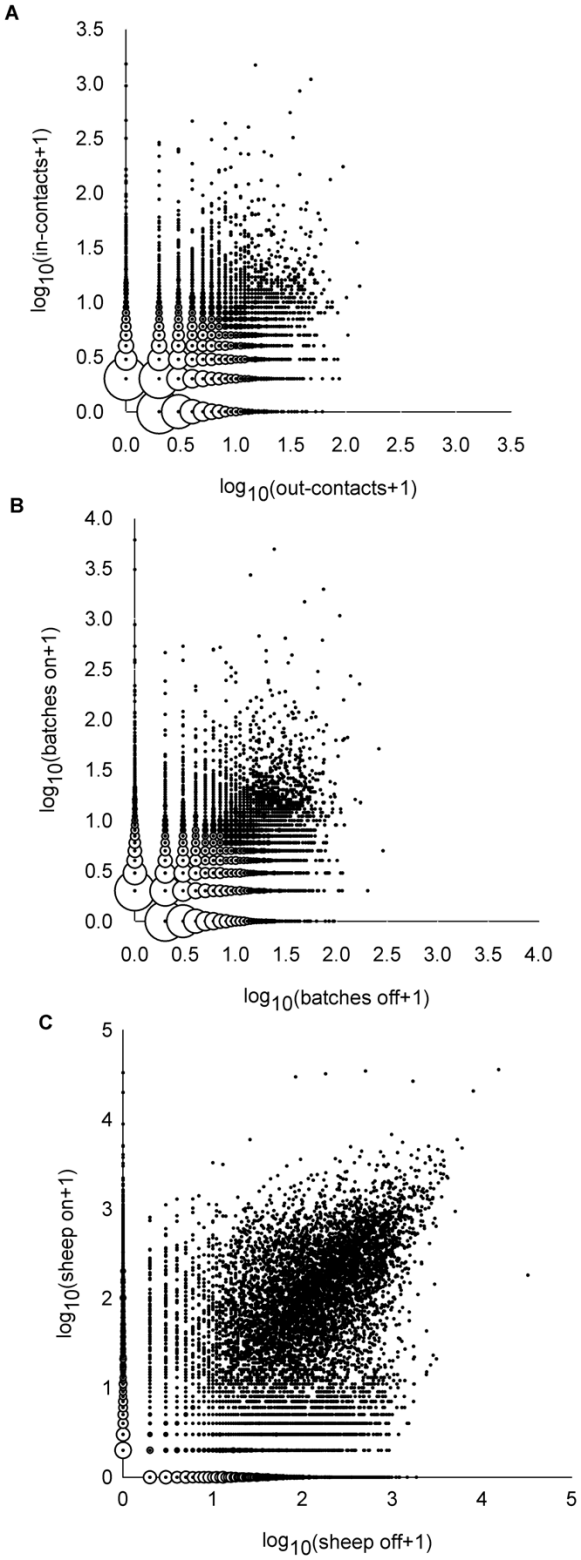
Co-distribution of sheep movements on and off Scottish farms 1 July 2006 to 30 June 2007.

The mean number of farm contacts per year was within the range 4.3 to 4.7 for the 4 years studied (Table 2A). The distributions of numbers of in-contacts (farms sheep were brought from) and out-contacts (farms sheep were sent to) made by individual farms in one year were highly over-dispersed (Figure 1A), with only a small fraction of the farms making large numbers of contacts. The variances in the numbers of in-contacts were much greater than that of out-contacts (Table 2A). The linear correlations between the numbers of annual in-contacts and out-contacts of the farms, *r_βinβout_*, were positive but weak over the 4 years studied (Pearson correlation coefficient +0.07 to +0.11, all *p*<0.001) (Table 2A and Figure 2A).

**Table 2:** Properties of contact matrices for Scottish farms via sheep movements.

Year	Mean of weighted contacts	Variance in-contacts	Variance out-contacts	Correlation in-contacts and out-contacts	Ratio of mean product in-contacts* out-contacts to mean contacts	Multiplicative effect of second order moments of network on the relative magnitude of *R*_0_
**A** Unweighted contacts between farms						
Year 1	4.6	1,303	48.4	+0.081	9.0	1.98
Year 2	4.7	1,290	50.1	+0.077	8.9	1.89
Year 3	4.4	827	44.7	+0.074	7.6	1.74
Year 4	4.3	663	46.4	+0.109	8.8	2.02
**B** Contacts weighted by numbers of batches moved between farms						
Year 1	7.4	29,894	148.3	+0.054	22.7	3.07
Year 2	7.8	31,204	172.2	+0.035	18.3	2.35
Year 3	6.9	7,751	120.8	+0.059	15.2	2.20
Year 4	6.7	5,831	124.3	+0.072	15.8	2.36
**C** Contacts weighted by numbers of sheep moved between farms						
Year 1	140.5	2,600,728	96,719	+0.200	855.3	6.09
Year 2	138.3	2,203,887	82,796	+0.183	704.0	5.09
Year 3	137.2	483,045	85,900	+0.360	670.9	4.89
Year 4	143.9	555,775	168,741	+0.303	789.5	5.48

The mean numbers of batches of sheep received by (or sent from) a farm in a year was in the range 6.7 to 7.8 over the 4 years (Table 2B). The variances in the numbers of batches received by a farm were much greater than that in the numbers of batches moved off (Table 2B and Figure 1B). The linear correlations between the numbers of batches moved on and off the farms in a year were slightly lower (Pearson correlation coefficient +0.04 to +0.07, all *p*<0.001) (Table 2B and Figure 2B) than the correlations between the numbers of annual in-contacts and out-contacts.

The mean numbers of sheep received by (or sent from) a farm in a year was in the range 137 to 144 per year (Table 2C). The variances in the numbers of sheep received by a farm were much greater than that in the numbers of sheep moved off (Table 2C and Figure 1C). The linear correlations between the numbers of sheep moved on and off the farms in a year were higher (Pearson correlation coefficient +0.18 to +0.36, all *p*<0.001) (Table 2C and Figure 2C) than the correlations between the numbers of batches moved on and off or between the numbers of annual in-contacts and out-contacts.

### Impact of network's moments on the magnitude of *R*_0_

To make the analyses relevant to diseases with different animal-level prevalence on affected farms the directed contact rate from farm *j* to farm *i* in a particular year, *a_ij_*, was defined in three ways: 1) present or absent (unweighted), 2) weighted by the number of batches of sheep moved (batch-weighted), and 3) weighted by the total number of sheep moved (animal-weighted). Model 1 is most appropriate for a highly transmissible infection with high animal-level prevalence, which would be likely to be transmitted via any sheep movement from farm *j* to farm *i*. Model 3 is most appropriate for a rare infection with low animal-level prevalence, for which the probability of transmission could be considered to depend linearly on the number of sheep moved farm *j* to farm *i*. Model 2 is an intermediate scenario, here represented by the farm contact weighted by the numbers of batches of sheep moved. For all three models, the contribution of heterogeneities in contact rates of the farms, second order moments of the farm contact matrix, to *R*_0_ was quantified as the ratio of the quantity calculated in Expression [3] in *Methods* to the mean farm contact rate.

Using unweighted *a_ij_* values (Model 1) the net contribution of second order moments of the contact network was to increase (from that contributed by the first order moment alone) the magnitude of *R*_0_ by up to a factor of 2 (Table 2A, *Column 7*). This contribution varied only slightly throughout the years of the study. Using *a_ij_* values weighted by numbers of batches moved between farms (Model 2) the net contribution of the second order moments of the contact network was to increase the value of *R*_0_ by a factor of more than 3 in year 1 but was lower, 2.20 to 2.36, in years 2 to 4 (Table 2B, *Column 7*). Using *a_ij_* values weighted by numbers of sheep moved between farms (Model 3) the net contribution of the second order moments was to increase the value of *R*_0_ by a factor of more than 6 in year 1 but was also lower, 4.89 to 5.48, in the years 2 to 4 (Table 2B, *Column 7*).

### Effectiveness of interventions targeted at the top-contributors to the magnitude of *R*_0_

**Figure 3:**
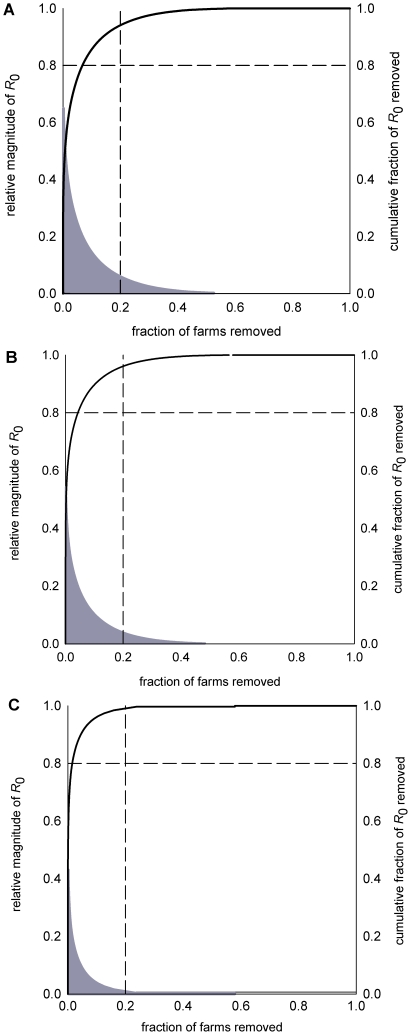
Contribution of individual farms to the magnitude of *R*_0_ 1 July 2006 to 30 June 2007.

The ‘20–80’ rule reflects that, in many situations, the potential for transmission of infection can be reduced by at least 80% by targeting just 20% of the members of population [2]. In the Scottish sheep farm network, removing the contribution of top 20% of farms most contributing resulted in at least 90% reduction in the magnitude of *R*_0_ in any of the 4 years studied regardless of how contacts were weighted (Figure 3 and Table 3, *Column 2*).

**Table 3:** Contribution to *R*_0_ of top 20% of farms identified from current year versus preceding year farm contact information.

Year	% reduction in *R*_0_ based on targeting top 20% of farms of current year	% reduction in *R*_0_ based on targeting top 20% of farms of preceding year
**A** Unweighted contacts between farms		
Year 1	93.6%	-
Year 2	93.7%	86.0%
Year 3	93.4%	84.8%
Year 4	94.1%	87.7%
**B** Contacts weighted by numbers of batches moved between farms		
Year 1	97.1%	-
Year 2	96.6%	91.2%
Year 3	96.2%	89.7%
Year 4	96.1%	90.7%
**C** Contacts weighted by numbers of sheep moved between farms		
Year 1	99.1%	-
Year 2	99.0%	93.5%
Year 3	98.9%	89.1%
Year 4	99.1%	90.0%

The magnitude of *R*_0_ was reduced by at least 80% in a given year for animal-weighted contacts if the contributions of 6.8% to 8.1% of farms were removed. These fractions were smaller for unweighted contacts, from 1.1% to 2.1% of farms, and, for batch-weighted contacts were from 2.2% to 4.3% of farms.

In practice, farm contact information from the preceding year may be more readily available than real-time data. In the Scottish sheep network the identities of the top 20% of farms contributing the most to the magnitude of *R*_0_ changed from year to year. For animal-weighted contacts approximately 70% of farms in the top 20% contributors to the transmission potential in a given year also appeared in this fraction the following year. This fraction was similar for batch-weighted contacts, but was 65% or less for unweighted contacts. When the contacts of 20% of farms most contributing to the magnitude of *R*_0_ in the preceding year were removed in the current year, the resulting reductions in the value of *R*_0_ were consistently smaller, and also more variable, compared with targeting the current year's top-contributors (Table 3, *Columns 3 and 4 versus Column 2*).

## Discussion

Although there have been numerous studies of contact networks as they relate to transmission of infectious diseases, very few of these investigate complete networks, and those that do have generally dealt with small populations [19]. Livestock movement databases allow analyses of large and complete networks, here, covering the entire population of Scottish sheep farms. Another feature of the majority of studies of contact networks is that they consider bi-directional contacts. Again, livestock movement databases are unusual due to explicit designation of uni-directional contacts where movement of livestock from farm *j* to farm *i* is associated with risk of disease transmission only in that direction [20]. This paper therefore provides information on the structure of contact networks and its relationship to the potential for spread of infectious diseases not readily available from studies of other populations.

The size of the giant weakly connected component of the network and relatively small number of movements from farms outside Scotland confirm that Scottish sheep farms can be regarded as a single population connected by sheep movements for the purposes of these analyses. The size of this giant component relative to the size of the network confirms that the Scottish sheep industry is inter-connected, in contrast, for example, to the commercial pig industries where animal movements are largely constrained within sub-networks [21], [22]. Notably, this large connected component emerges even though the contract matrix itself is very sparse (with approximately 0.03% non-zero entries in a year) reflecting that, on average, each farm moves sheep to or receives sheep from less than five other Scottish farms in a given year.

We can then use calculation of the basic reproduction number, *R*_0_, as a method to characterise the properties of the network of contacts between Scottish farms via movements of sheep and how these properties relate to the spread of infectious diseases within that population of farms. For a number of reasons however these calculations do not represent formal estimates of *R*_0_ for any specific infectious disease. First, as indicated in Expressions [1]–[3] in *Methods*, we generate relative, not absolute, measures of the magnitude of *R*_0_. Nor are the different contact formulations (unweighted, batch-weighted and individual animal-weighted farm contact) directly comparable amongst themselves (each being most relevant to certain disease scenarios, as discussed above). Secondly, we aggregate all movements over a one-year interval to provide a measure of relative contact rates for the farms. This does not account for temporal heterogeneities within the year, in particular marked seasonality in Scottish sheep movements; these can significantly affect *R*_0_ [23] and could influence the results reported here if temporal variations in contact rates were poorly correlated across the farms. Finally, although movement of livestock is an important route for the spread of many endemic livestock infections, it is typically not the only route; other routes of transmission of infections between farms may be relevant for specific applications, e.g. wildlife [24]–[27], insect vectors [28], fomites and visitors or over-the-fence contact [29], [30].

The size of the Scottish sheep farm network, the sizes of its giant weakly and strongly connected components, of its giant out-component, and the mean contact rates of a farm were broadly consistent across the 4 years (Table 1). However, there were differences in the contributions of the network's second order moments to the relative magnitude of *R*_0_ throughout the years using animal-weighted or batch-weighted contacts. Previous studies of contact networks have reported increases in the value of *R*_0_ associated with heterogeneities in contact rates between individuals [19]. Here we find that the size of such effects vary according to how the contacts are weighted.

However, the impact of second order moments on the magnitude of *R*_0_ is far less than might be anticipated from the very high variances in farm contact rates [2]. The explanation is that there is only a weak correlation between the movements on and movements off individual farms (Table 2). Nonetheless, because these correlations are positive (if negative, the effect would be to reduce *R*_0_, see Expression [2]) and the variances of contact rates are so high, the net effects are still substantial for all of the disease scenarios considered.

Given the importance of heterogeneities in farm contact rates in determining the magnitude of *R*_0_, it is apparent that targeting interventions at farms contributing the most to *R*_0_ is likely to be efficient. Interventions (e.g. pre-movement testing or, for some diseases, preventive vaccination) may reduce or eliminate the risk of disease transmission via livestock movement to or from individual farms. In practice, the 100% reduction in the susceptibility or infectiousness of individual farms is unlikely to be feasible.

Notably, information on contacts of farms in the preceding year was consistently slightly less valuable for identifying the 20% of farms to target in the current year (Table 3). This result presumably reflected some year-to-year variation in individual farms' contact patterns (Table 2). As to the processes underlying such variation, characterising the farms repeatedly or intermittently appearing in the 20% contributing the most to the potential for transmission of infections each year may provide further insights. We note that the contact patterns of farms can also be altered by changes to the legal restrictions on livestock movements.

The key conclusions arising from this work are as follows. First, second order properties of a contact matrix (i.e. those not quantifiable from knowledge of the mean contact rates alone) can have a substantial impact on the magnitude of *R*_0_, see also Anderson and May [3], Woolhouse *et al.* [2] and others. Here we quantify the impact that heterogeneities in contacts rates of farms have on the potential for transmission of infections of livestock in a farm population. Second, the way in which contacts are weighted or defined makes a very substantial difference to quantification of *R*_0_ and its components. When and how contacts should be weighted is relatively straightforward for livestock movements, perhaps less so for other kinds of ‘contact’ between individuals in a population. Third, contact matrices may vary through time not only in terms of contact rates of individual members of the population but also in terms of higher order properties, as has been reported previously for the UK cattle movement network [15] and observed here for the Scottish sheep movement network. The wider applicability of these conclusions depends on how representative the livestock farm networks are of contact networks in general, but we conjecture that similar issues will arise in many other contexts.

## Methods

### Sheep movement data

Records of sheep movements among Scottish holdings from 2003 to 2007 were obtained from the Scottish Animal Movement System (SAMS), operated by the Scottish Government. The aim was to consider a recent period of several years not interrupted by major restrictions of livestock movements. The SAMS system was launched during 2002. An outbreak of foot-and-mouth disease in Surrey, England led to restrictions of livestock movements in Scotland from 3 August 2007 to 31 December 2007. Therefore the period of 4 consecutive years from 1 July 2003 to 30 June 2007 was chosen for the analysis.

The SAMS records were processed using the Python programming language and then in SAS® 9.1.3 software for Windows (SAS Institute Inc., Cary, NC, USA). Up-to-date lists of sheep markets, show-grounds, abattoirs and other industry units registered in Scotland were collated with help from Livestock Traceability Policy Branch, Animal Health and Welfare Division, the Scottish Government and from Animal Health agency in Scotland. The data were processed, including definitions of types of holdings and movements, as previously described [31]. In short, and pertinent to these analyses, the vast majority (>99%) of the SAMS entries for sheep 2003 to 2007 were logical movement records, and the number of sheep movements not reported to SAMS during this period was believed to be low.

Four one-year intervals were analyzed: Year 1, 1 July 2003 to 30 June 2004; Year 2, 1 July 2004 to 30 June 2005; Year 3, 1 July 2005 to 30 June 2006; and Year 4, 1 July 2006 to 30 June 2007. The June/July dividing date precedes the major annual movement of sheep in the autumn. Seasonality in sheep movement patterns is not considered further in these analyses.

A farm was included in a year's analysis if it either sent or received sheep from another Scottish farm directly or via a Scottish livestock market during that year (movements to and from designated show-grounds and to slaughter were excluded).

During the period of study, the sheep identification and traceability regulations in Scotland did not require specification of individual animals in the movement documents (the Sheep and Goats Movement Interim Measures Scotland Order 2002 and Amendments; the Sheep and Goat Identification and Traceability Scotland Regulations 2006 and Amendments). Therefore the length of stay of an individual sheep on a given farm could not be determined. The legally required standstill period was 13 days, i.e. no sheep should have been moved off the farm earlier than 13 days after a sheep on-movement unless to slaughter, although certain categories of movements were exempt from the standstill. Sheep housed on mixed livestock farms were also subject to standstill after an on-movement of cattle (13 days), pigs (20 days) or goats (13 days).

The focus of these analyses was the network of Scottish farms via movements of sheep. For this purpose the network was treated as closed and movements outside Scotland were ignored. In practice, cross-border movements onto Scottish farms, primarily from England and Wales, did occur, but at low rates (<2% of movements onto Scottish farms during the study period). Movements off Scottish farms to locations outside Scotland were much more frequent, but are not relevant here.

Within Scotland, the majority of sheep movements between the farms (>80% in each of the 4 years analysed) occurred via Scottish livestock markets. Since we considered a relatively long time period (full year) and diseases with prolonged periods of infectivity of affected animals, we assumed the potential for disease transmission during brief stays at markets to be negligible compared to that on the farms (noting that this assumption would not hold for acute infections which are transmitted over short time scales). Therefore, we treated any indirect movement from farm *j* to farm *i* via a market as equivalent to a direct movement from farm *j* to farm *i*.

### Giant network components

Connectedness of the farm network in each of the 4 years was evaluated by calculating the giant strongly connected component (GSCC), the giant weakly connected component (GWCC) and the giant out-component (GOC) of the network. The GSCC and GWCC were calculated with Tarjan's algorithm [32] implemented in C++. The GOC was calculated by choosing a farm from the GSCC and performing a depth-first search excluding cycles to identify every farm reachable from the chosen farm by a direct path; this was implemented in C++. For a given year, the GSCC encompassed all farms linked by bi-directional contacts; the GOC encompassed the GSCC plus all farms reachable from the farms in GSCC by a direct path (‘sinks’); and the GWCC encompassed the GSCC plus all farms connected to the farms in the GSCC by any uni-directional contact (both ‘sources’ and ‘sinks’).

### Definitions of contact between farms

Let *a_ij_* be the directed contact rate from farm *j* to farm *i* in a particular year. We assign values to *a_ij_* in one of three ways. 1) contact scored as 0 (no movement of sheep from farm *j* to farm *i*) or 1 (any movement of sheep from farm *j* to farm *i*); 2) as (1) but weighted by the number of batches of sheep moved from farm *j* to farm *i* (noting that this is equivalent to the frequency of contact from *j* to *i*); and 3) as (1) but weighted by the number of sheep moved from farm *j* to farm *i*. Model 1 is most appropriate for an infection with high animal-level prevalence on affected farms (i.e. likely to be transmitted via any movement of sheep between farms). Model 3 is most appropriate for a rare infection with low animal-level prevalence (so the probability of transmission can be considered to depend linearly on the number of sheep moved between farms). Model 2 is intermediate between 1 and 3.

### Calculating contributions of network's first and second order moments to the magnitude of *R*_0_

For all three disease models discussed above, the in-contact rate for farm *i* is *β^i^*_in_ = Σ*_j_a_ij_*, and the out-contact rate is *β^i^*_out_ = Σ*_j_a_ji_*. *R*_0_ is related to the mean contact rate. In a closed network Σ*_i_β^i^*_in_ = Σ*_i_β^i^*_out_ and, if we were to assume that there was no variation in individual contact rates then:
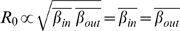
(1)More generally, *R*_0_ is further influenced by the second order moments of the contact matrix. We denote the standard deviation of in-contact rates as σ(*β*_in_), the standard deviation of out-contact rates as σ(*β*_out_), and the Pearson product-moment correlation coefficient between in-contact rates and out-contact rates as *r_βinβout_*. As previously shown [20], *R*_0_ (ignoring higher order properties of the network) is a function of these terms as follows:
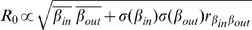
(2)Therefore, non-zero variances of *β*_in_ and *β*_out_ can increase *R*_0_ if *β*_in_ and *β*_out_ are positively correlated. Expression [2] can be written in terms of the product of *β*_in_ and *β*_out_, denoting the number of farms in the network as *N*, this is:
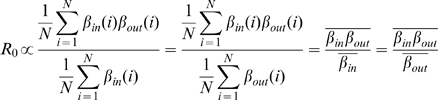
(3)where *β*_in_(*i*) and *β*_out_(*i*) refer to in- and out-contact rate, respectively, for farm *i*.

The contribution of second order moments of the farm contact matrix to *R*_0_ was evaluated as the ratio of the quantity calculated in Expression [3] to the quantity calculated in Expression [1].

Quantities [1] and [3] were calculated for the contact matrices where contacts between farms were weighted according to each of the three scenarios of the animal-level prevalence of the disease.

### Calculating contributions of individual farms to the magnitude of *R*_0_

For each of the 4 years and three disease scenarios, we assumed that contacts of a farm were non-infectious or absent (setting *β*_in_ *β*_out_ = 0 for the farm) and re-calculated the contribution of the first and second order moments of the network to the magnitude of *R*_0_. The resultant quantity evaluated individual contribution of the farm to the magnitude of *R*_0_ and allowed ranking the farms in the order of their contribution.
